# SIRT1 Promotes Breast Cancer Stem Cell-Associated Properties and Represents a Potential Therapeutic Target

**DOI:** 10.3390/ijms27188226

**Published:** 2026-09-15

**Authors:** Jianmin Ding, Baoxiang Guan, Xuejun Fan, Ningyan Zhang, Eva Sevick, Songlin Zhang

**Affiliations:** 1 Department of Pathology & Immunology, Baylor College of Medicine, One Baylor Plaza, Houston, TX 77030, USA; jianmin.ding@bcm.edu; 2 Department of Genitourinary Medical Oncology, The University of Texas MD Anderson Cancer Center, Houston, TX 77030, USA; bguan@mdanderson.org; 3 Texas Therapeutics Institute, Brown Foundation Institute of Molecular Medicine, McGovern Medical School, University of Texas Health Science Center at Houston, 1825 Pressler Street, Houston, TX 77030, USA; xuejun972002@hotmail.com (X.F.); ningyan.zhang@uth.tmc.edu (N.Z.); 4 Center of Molecular Imaging, Brown Foundation Institute of Molecular Medicine, McGovern Medical School, University of Texas Health Science Center at Houston, 1825 Pressler Street, Houston, TX 77030, USA; eva.sevick@uth.tmc.edu

**Keywords:** breast cancer, cancer stem cell (CSC), breast cancer stem cell (BCSC), epithelial–mesenchymal transition (EMT), SIRT1, SIRT1 inhibitor, TGF-β, Wnt/β-catenin pathway, DVL

## Abstract

Breast cancer stem cells (BCSCs) drive tumor progression, metastasis, and therapeutic resistance. Sirtuin 1 (SIRT1) has been implicated in stem cell regulation, but its context-dependent role in BCSC maintenance and the underlying molecular mechanisms remain poorly defined. SIRT1 expression was evaluated in human breast cancer specimens. Pharmacologic and genetic SIRT1 inhibition was assessed in breast cancer cell lines and orthotopic xenograft models to determine effects on stemness, epithelial–mesenchymal transition (EMT), metastasis, and chemoresistance, with emphasis on Wnt/β catenin signaling. SIRT1 was overexpressed in breast cancer and correlated with higher tumor grades. SIRT1 inhibition reduced BCSC markers (CD44, ALDH1), stemness genes (NANOG, SOX-2), mammosphere formation, EMT, and invasion. In vivo, SIRT1 inhibition suppressed tumor growth, blocked lymphatic metastasis, and delayed cisplatin resistance. Mechanistically, these effects were mediated by downregulation of Disheveled 3 (DVL3) and attenuation of Wnt/β catenin signaling. SIRT1 is associated with the maintenance of BCSC-related properties, EMT, and activation of the Wnt/β-catenin pathway in breast cancer. Targeting SIRT1 may represent a potential therapeutic strategy to suppress tumor progression and improve treatment response.

## 1. Introduction

Breast cancer is the second most-commonly diagnosed cancer overall, and the most-common cancer in women globally [1]. According to the International Agency for Research on Cancer (IARC), on average 1 in 20 women worldwide will be diagnosed with breast cancer in their lifetime. If current trends continue, by 2050 there will be about 3.2 million new breast cancer cases per year globally [2]. American Cancer Society estimates for 2025 are about 316,950 new invasive breast cancer cases in women, 59,080 cases of ductal carcinoma in situ (DCIS) in women, and 42,170 deaths [3].

Although advances in early detection and therapy have significantly improved overall survival in breast cancer, the recent mRNA vaccine is a milestone in triple-negative breast cancer [4]. Disease recurrence and metastasis remain major causes of mortality. These adverse outcomes are largely attributed to a small subpopulation of breast cancer stem cells (BCSCs) that survive initial treatment, evade eradication, and drive tumor relapses and drug resistance [5,6]. BCSCs possess stem-like properties, including self-renewal, differentiation, and tumor-initiating capacity, and were first identified as CD44^+^/CD24^−/low^ cells in breast cancer tissues by Al-Hajj et al. [7].

BCSCs are intrinsically resistant to conventional therapies due to quiescence, enhanced DNA repair, and activation of pro-survival pathways such as Wnt/β-catenin, Notch, and Hedgehog [8]. They are characterized by CD44^+^/CD24^−/low^, high ALDH1 activity, and EMT markers such as vimentin and ZEB1/2, with reduced E-cadherin expression [9,10]. EMT promotes invasion and metastasis, while CD44^+^/CD24^−/low^ and ALDH1-positive populations are enriched in stem-like, tumorigenic, and metastatic cells [11,12,13,14,15].

BCSCs are regulated by developmental pathways, particularly Wnt/β-catenin signaling, whose increased activity promotes self-renewal, metastasis, and chemoresistance [16,17,18]. Wnt/β-catenin and NF-κB signaling regulate stemness factors including SOX-2, NANOG, OCT4, KLF4, c-MYC, and ALDH1A1, supporting BCSC maintenance and therapeutic resistance [17,19]. Thus, targeting BCSCs and their regulatory networks may prevent relapses and improve patient outcomes [20].

Sirtuin 1 (SIRT1) is a class III histone deacetylase (HDAC) that deacetylates both histone and non-histone proteins, thereby regulating gene transcription and protein function. SIRT1 plays important roles in cell growth, apoptosis, senescence, and tumorigenesis [21,22]. Interestingly, SIRT1 exhibits dual effects in carcinogenesis, functioning either as a tumor suppressor [23,24] or as a tumor promoter [25,26,27,28]. The role of SIRT1 in breast cancer remains complex and controversial, as its activity can influence both oncogenic and tumor-suppressive pathways, leading to divergent outcomes in different studies.

In the context of cancer stem cells, SIRT1 has been shown to play essential roles in the maintenance and differentiation of various cancer stem cells, including glioma [29], colorectal [30], pancreatic [31], chronic and acute myeloid leukemia [32], and BCSCs [33]. The regulation of SIRT1 in cancer stem cells involves several mechanisms, such as modulating key factors within stem cell activation signaling pathways, including Bcl-2 [34], P53 [35], Nf-κB [36], and Wnt/β-catenin [37], or interacting with microRNAs that influence cancer stem cell behavior [30]. Therefore, SIRT1 is considered to play a critical role in tumorigenesis and exhibits a strong association with cancer stem cell regulation.

Breast cancer cell lines provide established in vitro models to investigate BCSC properties and signaling pathways regulating self-renewal, stemness, and therapeutic resistance, while xenograft models enable in vivo validation of their effects on tumor initiation, growth, and therapy response. The triple-negative breast cancer (TNBC) cell lines MDA-MB-231 and MDA-MB-468 are particularly useful models for studying BCSC-related phenotypes because they exhibit prominent stem-like properties. We hypothesized that SIRT1 promotes breast cancer and therefore examined SIRT1 expression and BCSC characteristics in human breast cancer tissues, investigated its role and underlying signaling pathways in breast cancer cell lines, and used xenograft models to evaluate the effects of SIRT1 inhibition on tumor growth and metastatic potential.

## 2. Results

### 2.1. SIRT1 Expression Is Elevated in Human Breast Cancer and Correlates with Tumor Grade, Vimentin and E-Cadherin Expression, and Other EMT/Stem Cell Markers

A total of 35 breast cancer specimens and 2 benign breast tissue specimens were obtained from patients who underwent surgical resection, with approval from the Institutional Review Board (IRB, HSC-MS-13-0023). For analyses involving tumor type and stage, two breast cancer cases with other types and one case with an unknown stage were excluded, resulting in 32 evaluable breast cancer cases. Clinicopathological characteristics, including histological type, tumor grade, T stage, hormone receptor status, and axillary lymph node involvement, are summarized in Table 1. Immunohistochemical (IHC) staining was performed to assess the expression of SIRT1 and a panel of EMT markers (Vimentin, E-cadherin, Snail, and Twist) in both tumor and benign tissue sections. Notably, the *p* value revealed that SIRT1 expression was not significantly correlated with tumor type (*p* = 0.27) or tumor stage (*p* = 0.9) but was positively associated with tumor grade (*p* = 0.046).

**Figure 1:**
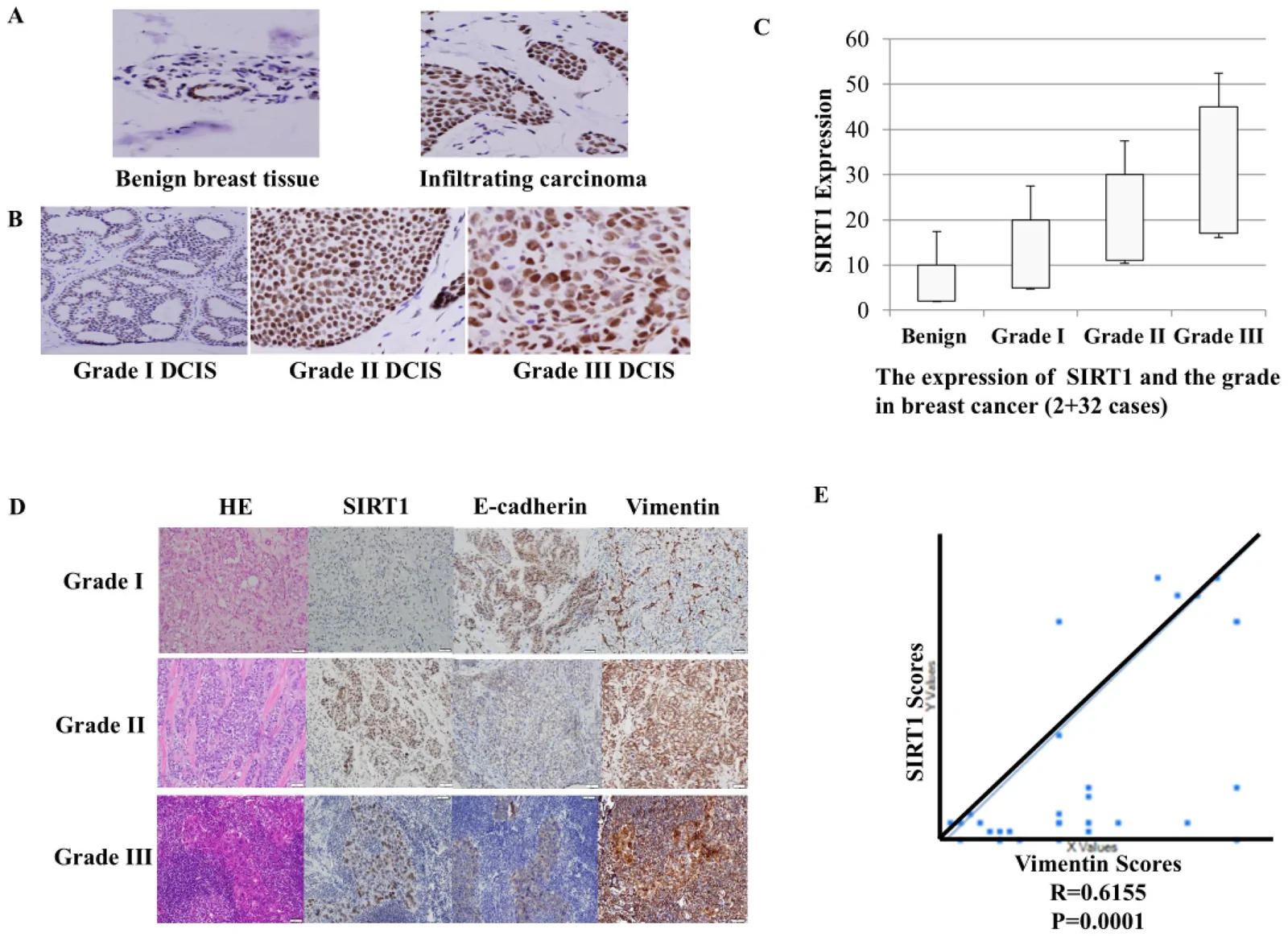
SIRT1 expression in Breast cancer significantly correlates with breast cancer grades and some stem cell markers. (**A**) Immunohistochemical (IHC) analysis of SIRT1 expression in benign breast tissue (left) and invasive carcinoma (right). (**B**) Representative SIRT1 staining in Grade I DCIS (left), Grade II DCIS (middle), and Grade III DCIS (right). (**C**) Quantification of SIRT1 expression across different histological grades (benign, number = 2; Grade I, number = 5; Grade II, number = 10; Grade III, number = 17, total, 2 + 32 cases). SIRT1 staining was scored using the H-score method (intensity 0–3 × percentage of positive cells), with possible scores ranging from 0 to 300. The mean H-scores were 10, 20, 30, and 45, respectively. (**D**) Representative IHC images showing co-expression of SIRT1, E-cadherin, and vimentin across tumor grades. Scale bar, 50 µm. (**E**) Correlation analysis between SIRT1 and vimentin expression (Pearson correlation, R = 0.6155, *p* = 0.0001). Statistical significance was determined using Chi-square and Pearson correlation tests.

SIRT1 expression was markedly elevated in most of the breast cancer specimens (21/33, 63.6%, Table 1). In ductal carcinoma in situ (DCIS), SIRT1 staining was predominantly nuclear and increased progressively with tumor grade, weak in grade I, moderate in grade II, and strong in grade III DCIS (Figure 1A,B). Quantitative analysis showed that the proportion of SIRT1-positive cases rose from 10% in grade I to 42% in grade III tumors (*p* = 0.046, G1/2 vs. G3; Figure 1C). No significant associations were found between SIRT1 expression and tumor histological type (IDC vs. ILC), T stage (T1 vs. T2/3), hormone receptor status (ER+, HER2+, or triple negative), or axillary lymph node metastasis.

To investigate the link between SIRT1 and BCSC and epithelial–mesenchymal transition (EMT), immunohistochemistry was scored semi-quantitatively using H-scores. Low-grade breast carcinoma showed strong membranous E-cadherin, weak vimentin expression, and low-level SIRT1 expression, whereas high-grade breast cancer displayed reduced E-cadherin, increased vimentin, and high-level SIRT1 expression (Figure 1D). Correlation analysis revealed a positive correlation between SIRT1 and vimentin expression (r = 0.6155, *p* < 0.01; Figure 1E); high-grade breast cancers showed decreased E-cadherin expression, but there was no statistical significance between SIRT1 and E-cadherin expression. All 33 breast cancers tested were negative for OCT-4, and Nanog showed some cytoplasmic staining in some cases but no nuclear positivity. Multiplex immunohistochemistry further revealed enrichment of breast cancer stem cell (BCSC) markers in high-grade tumors. Colocalization of ALDH1a and CD44 was significantly higher in grade III tumors compared with grade I/II tumors (56% vs. 7%, *p* = 0.007). Similarly, ALDH1a co-expression with CD133 and SOX-2 was more frequently observed in high-grade tumors (72% vs. 21%, *p* = 0.01). These findings indicate that elevated SIRT1 expression is associated with tumor grade, EMT features, and enrichment of BCSC populations in human breast cancer.

### 2.2. Pharmacologic Inhibition of SIRT1 Reduces BCSC-like Populations and Suppresses Stemness in Breast Cancer Cell Lines

**Figure 2:**
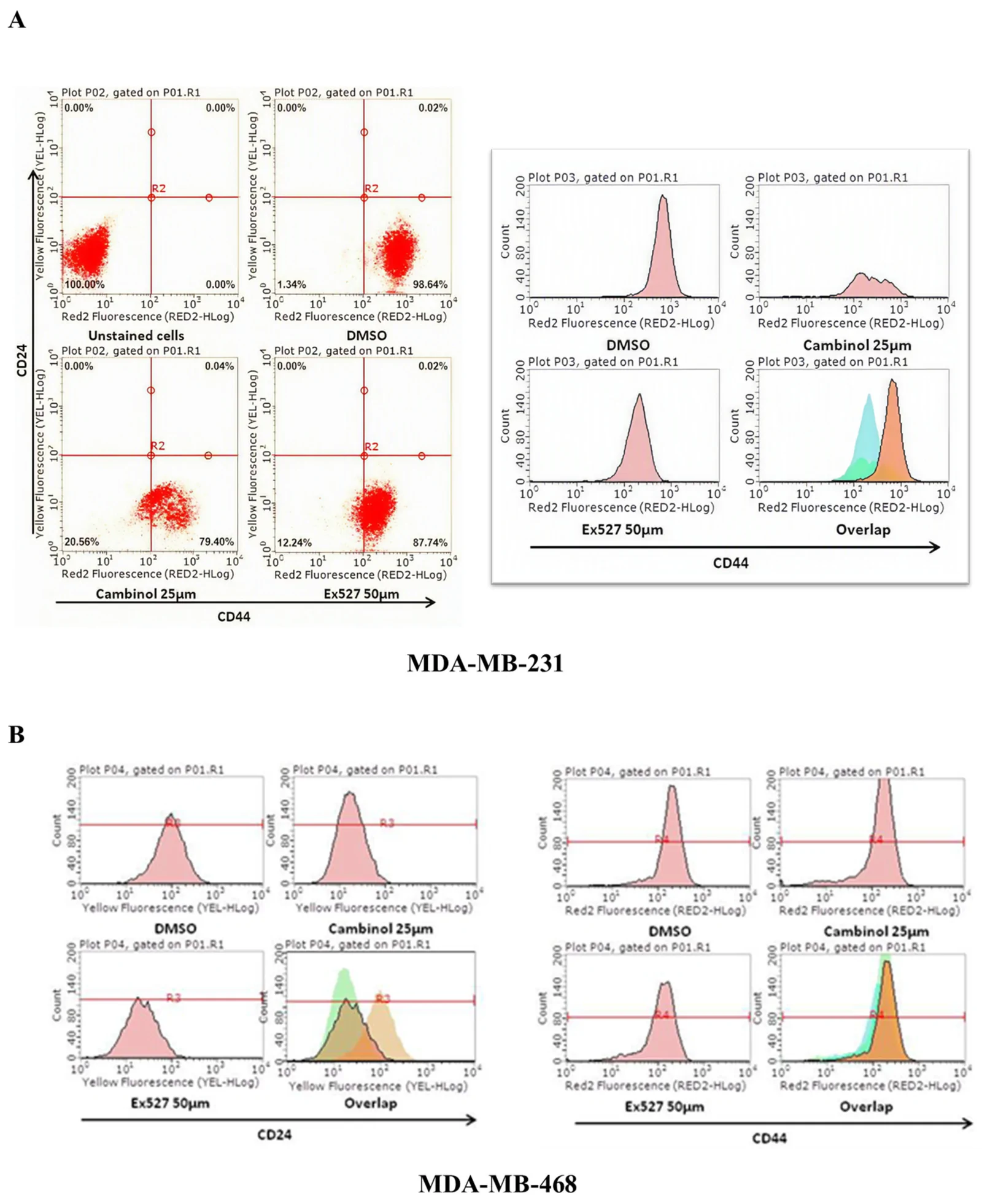

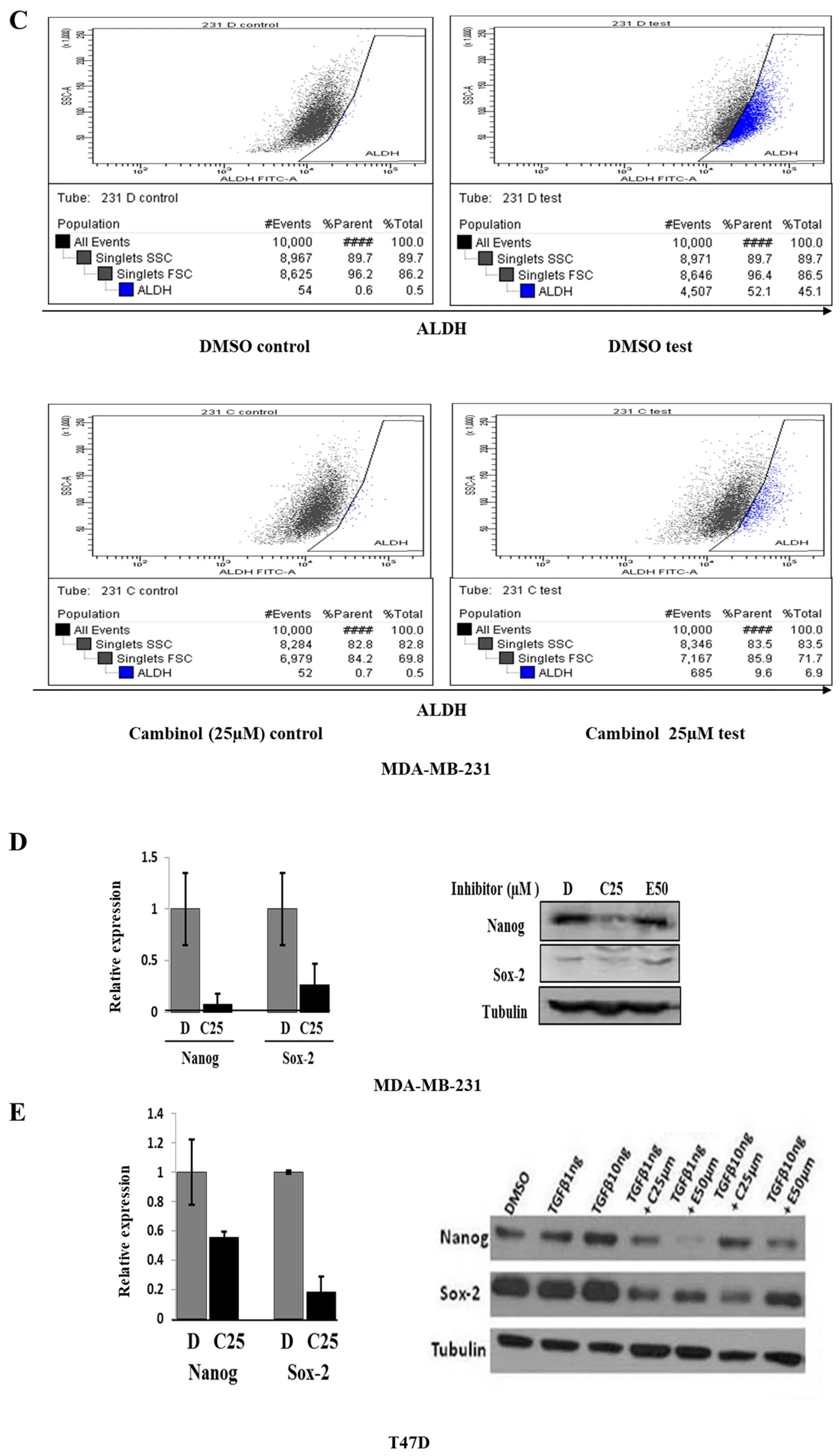
SIRT1 inhibitors alter CSC-associated phenotypes in breast cancer cell lines. (**A**) Representative flow cytometry plots showing the CD24/CD44 phenotype of MDA-MB-231 cells. Cells were treated with DMSO, cambinol (25 μM), or EX527 (50 μM) for 24 h. The dot plot (left) and histogram (right) show CD44 fluorescence intensity in untreated, DMSO-, cambinol-, and EX527-treated cells. (**B**) Histogram overlays showing CD24 (left) and CD44 (right) fluorescence intensity in MDA-MB-468 cells treated with DMSO, cambinol (25 μM), or EX527 (50 μM) for 24 h. (**C**) ALDH enzymatic activity was assessed using the ALDEFLUOR^®^ assay. Representative flow cytometry plots show the ALDH-positive cell populations in DMSO-treated cells (upper) and cambinol-treated cells (lower). The percentage of ALDH-positive cells is indicated in the respective plots. The symbols # and #### shown in the flow cytometry plots indicate event count information and N/A, respectively, as automatically displayed by the flow cytometry analysis software. (**D**) In MDA-MB-231 cells, qPCR analysis (left) showed that treatment with the SIRT1 inhibitor cambinol (C25, 25 μM) decreased the expression of the stem cell-associated genes Nanog and Sox2 compared with the DMSO control. Western blot analysis (right) showed reduced Nanog and Sox2 protein levels following cambinol treatment. (**E**) In T47D cells, qPCR analysis (left) showed that cambinol (C25, 25 μM) decreased the expression of Nanog and Sox2 compared with DMSO control. Western blot analysis (right) examined Nanog and Sox2 protein levels following treatment with TGF-β1 (10 ng/mL) alone or in combination with cambinol (C25, 25 μM) or EX-527 (E50, 50 μM). Tubulin was used as a loading control. Three independent experiments were performed, and representative data are shown.

To investigate whether SIRT1 inhibition blocks BCSCs, the SIRT1inhibitor cambinol (25 µM) [38] or EX527 (50 µM) [39] was used to treat breast cancer cells. Because BCSCs possess CD44^+^/CD24^−/low^ and Aldehyde dehydrogenase (ALDH) positive properties, we used either of the Sirt1 inhibitors to treat breast cancer cells and examine the expression of BCSC markers; the results are shown in Figure 2.

To investigate the BCSCs population, we assessed CD44/CD24 surface markers and ALDH-specific antibody in breast cancer cell lines (MDA-MB-231, MDA-MB-468, and T-47D). Following SIRT1 inhibitor treatment with 25 µM cambinol or 50 µM EX-527.

Flow cytometry analysis revealed that MDA-MB-231 cells, which are CD44^+^/CD24^−/low^ and representative of high-grade triple-negative breast cancer, exhibited a significant reduction in CD44 expression after 24 h of SIRT1 inhibitor treatment (Figure 2A). In MDA-MB-468 cells, which co-express CD44 and CD24, SIRT1 inhibition led to a pronounced decrease in CD24 expression and a modest reduction in CD44 levels (Figure 2B). In contrast, T-47D cells, a non-triple-negative, non-high-grade line, showed minimal CD44 expression and were largely unaffected by SIRT1 inhibition. ALDH activity was assessed using the ALDEFLUOR^®^ assay. DMSO-treated cells showed 45% ALDH^+^ cells (upper panel), whereas cambinol treatment reduced the proportion of ALDH^+^ cells to 6.9% (lower panel). Data are representative of three independent experiments. (Figure 2C). All experiments were repeated at least twice, yielding consistent results. These findings indicate that SIRT1 activity supports the maintenance of BCSC-associated surface markers, particularly in triple-negative breast cancer cell lines.

Consistent with these findings, quantitative RT PCR analysis revealed that SIRT1 inhibition significantly downregulated transcription of stemness-associated genes, including NANOG and SOX-2, in MDA MB 231 cells. Corresponding reductions in protein expression were confirmed by immunoblotting. In T-47D cells, due to their robust response to TGFβ1 stimulation [40] compared with triple-negative MDA-MB-231 cells, the cells were used for subsequent TGFβ1 experiments. Western blot analysis confirmed that SIRT1 inhibitors reduced Nanog and SOX-2 protein levels in T-47D cells, consistent with the qRT-PCR results (Figure 2D,E). These results demonstrate that SIRT1 activity is required for maintenance of BCSC-associated markers and transcriptional programs.

### 2.3. SIRT1 Inhibition Suppresses EMT, Self-Renewal, and Invasion in Breast Cancer Cells

**Figure 3:**
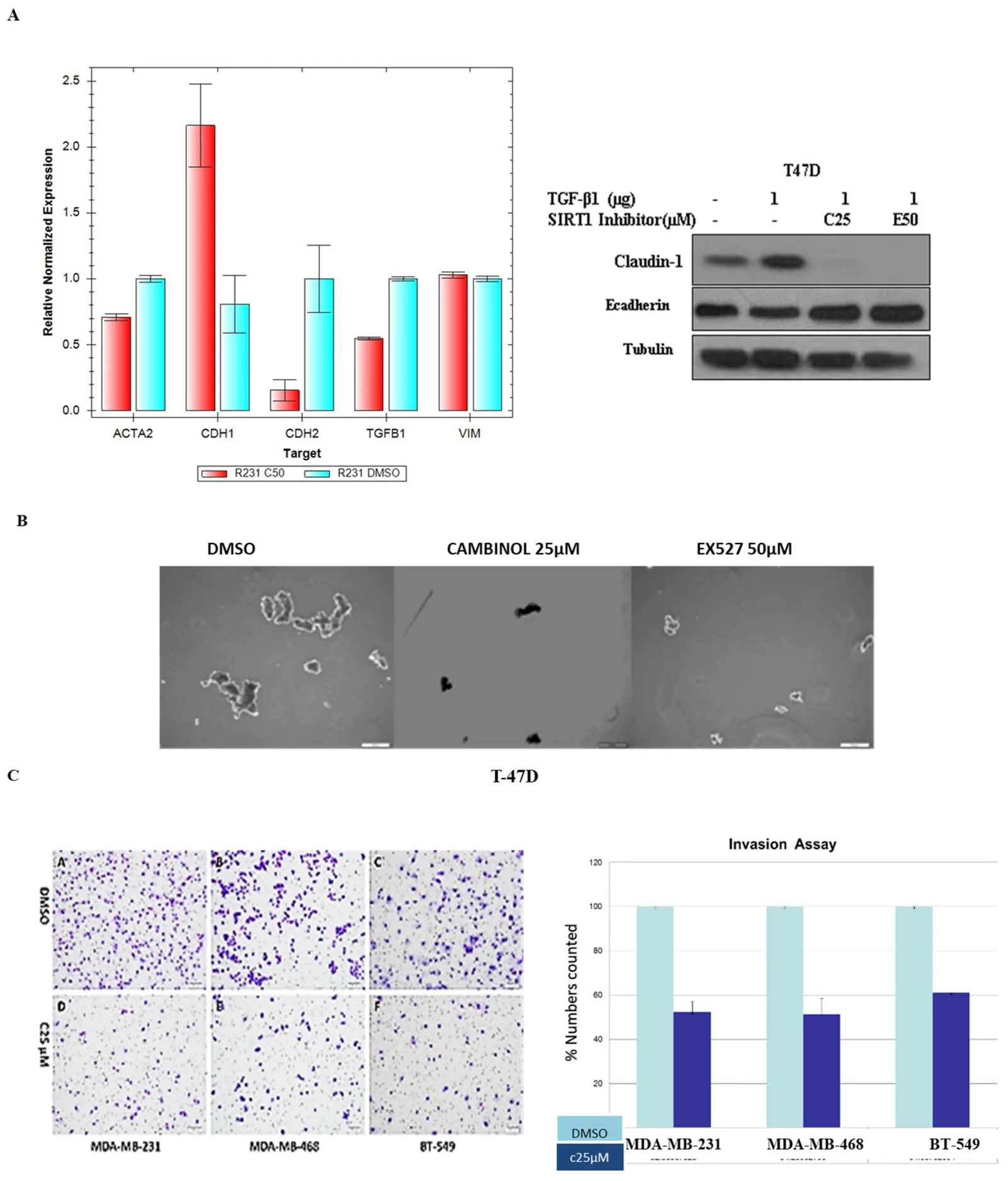
SIRT1 inhibition suppresses EMT, self-renewal, and invasion in breast cancer cells. (**A**) EMT-related molecular markers, including ACTA1 (actin alpha 1), CDH1 (cadherin 1), CDH2 (cadherin 2), TGFB1 (transforming growth factor beta 1), and VIM (vimentin), were determined in T47D cells. (**B**) Mammosphere formation assays were performed in T47D cells. Mammospheres with diameters > 75 μm were counted to quantify sphere formation. For the differentiation assay, individual mammospheres were cultured for 10–12 days, photographed, and colonies with diameters > 75 μm were quantified. (**C**) SIRT1 inhibition suppresses invasion in breast cancer cells. Cell invasion assays were performed using Matrigel™-coated 6-well Transwell inserts with an 8-μm pore size. MDA-MB-231, MDA-MB-468, and BT-549 cells were seeded in the upper chambers in serum-free DMEM, while the lower chambers contained DMEM supplemented with 10% FBS. DMSO or cambinol (25 μM) was added to the culture medium as indicated. After 24 h of incubation at 37 °C in 5% CO_2_, invaded cells were fixed, stained with Diff-Quik (IMEB Inc., San Marcos, CA, USA), and quantified by counting five randomly selected fields per insert. Three independent inserts were analyzed for each condition. (A,D) MDA-MB-231 cells; (B,E) MDA-MB-468 cells; (C,F) BT-549 cells. (A–C) DMSO treated groups; (D–F) cambinol (25 μM) treated groups.

Given the overlap between BCSC properties and EMT, we next examined the effect of SIRT1 inhibition on EMT markers and functional behaviors. In T47D cells stimulated with TGF β1, treatment with cambinol significantly inhibited the induction of mesenchymal markers, including vimentin, N cadherin, and smooth muscle actin, while restoring epithelial marker expression such as E cadherin and claudin 1 (Figure 3A).

Functional assays further demonstrated that SIRT1 inhibition impaired self-renewal and invasion. Mammosphere formation assays showed a significant reduction in both the number and size of spheres formed by T47D cells following treatment with cambinol or EX 527 (Figure 3B), indicating loss of self-renewal capacity. In Matrigel invasion assays, cambinol treatment significantly suppressed invasion of triple-negative breast cancer cell lines MDA-MB-231, MDA-MB-468, and BT-549 by 39–49% compared with controls (Figure 3C). Collectively, these data indicate that SIRT1 supports EMT-associated phenotypes, self-renewal, and invasive behavior in breast cancer cells.

### 2.4. SIRT1 Inhibition Suppresses Tumor Growth, Lymphatic Metastasis, and Chemoresistance In Vivo

**Figure 4:**
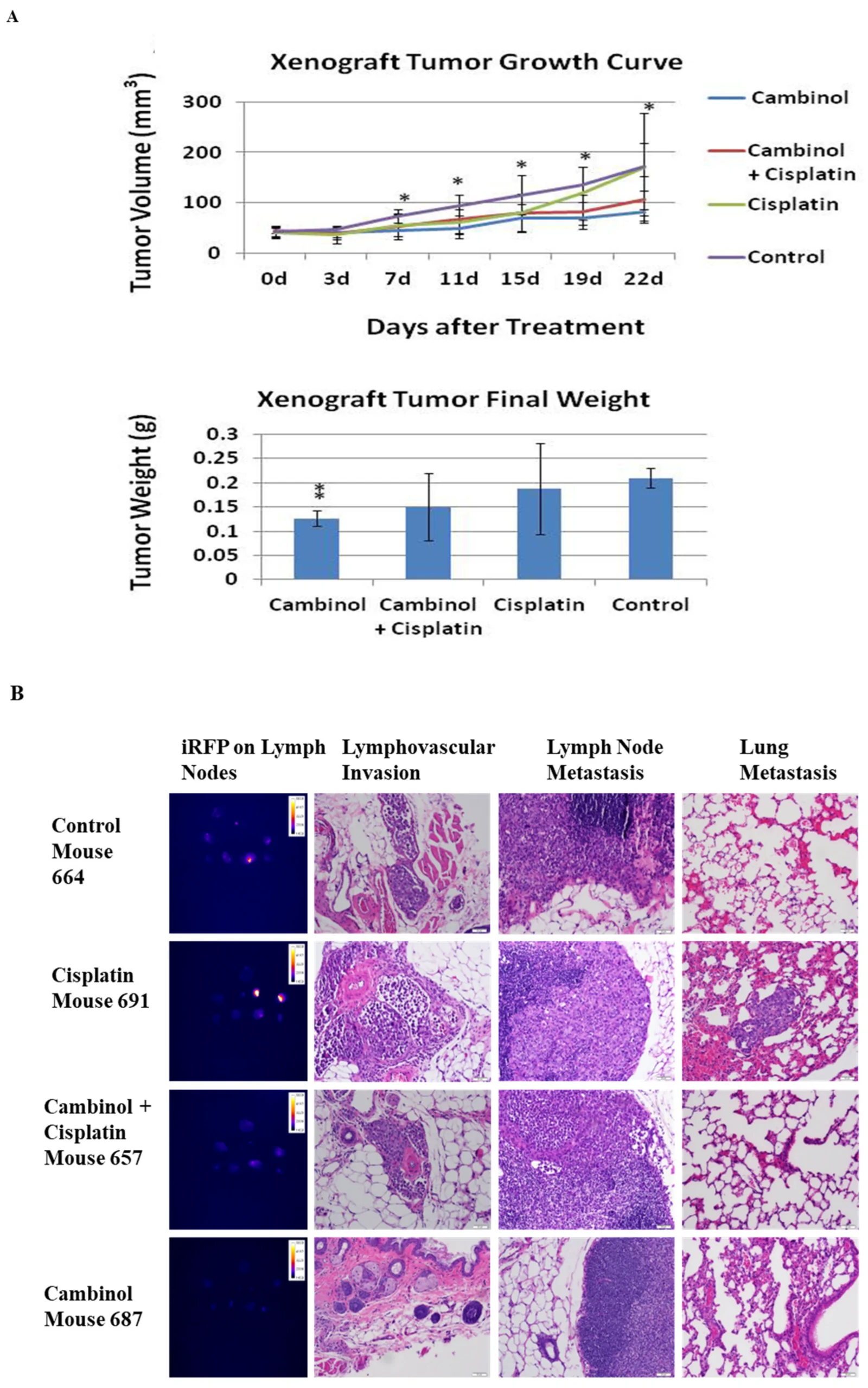

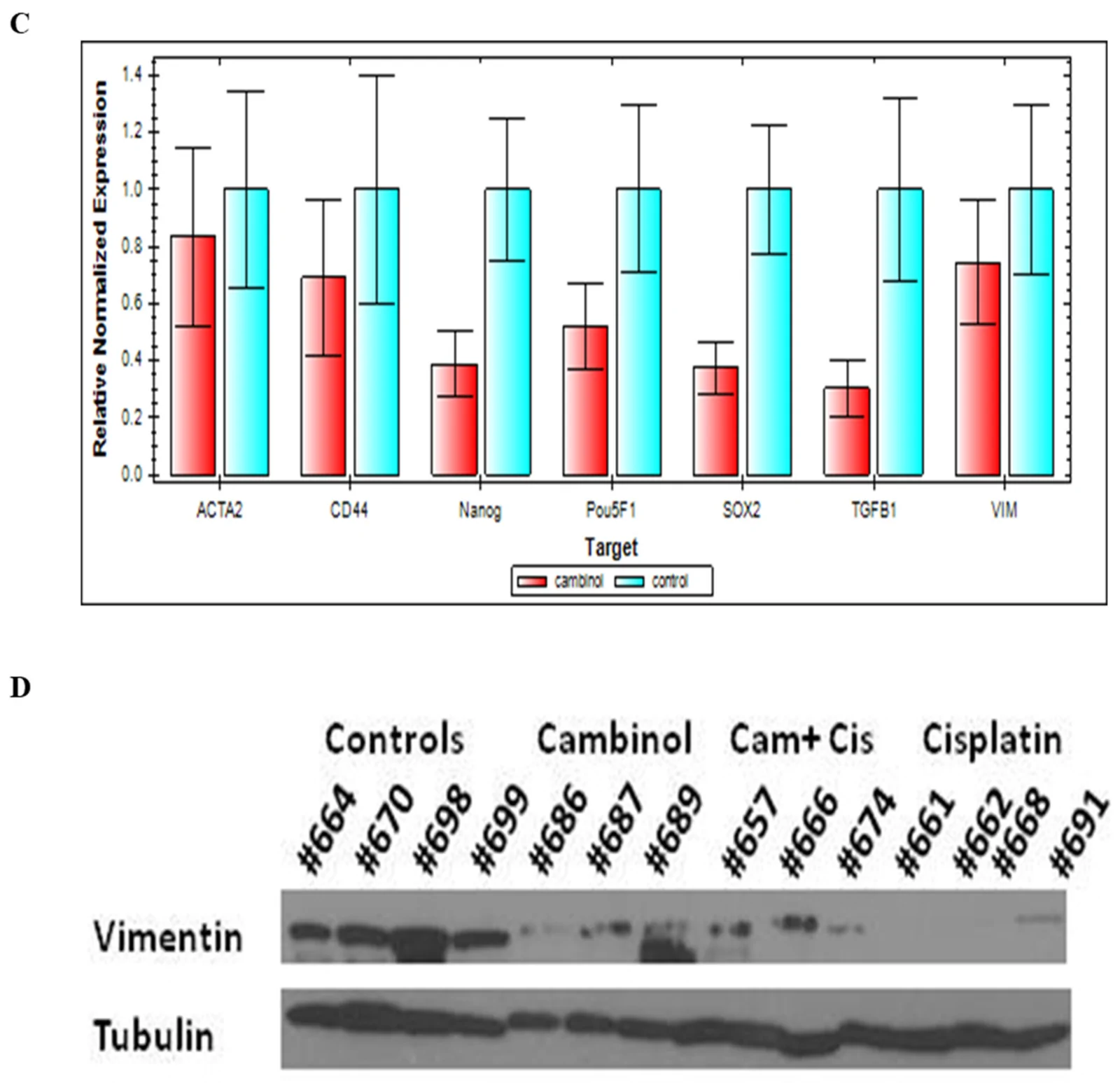
SIRT1 inhibition suppresses tumor growth and lymphatic metastasis in vivo. (**A**) Tumor growth and weight (* *p* < 0.05, ** *p* < 0.01). (**B**) Lymphatic spread. iRFP imaging showed lymphovascular invasion, lymph node metastasis and lung metastasis. (**C**) SIRT1 inhibition reduces BCSC and EMT-related gene expression. qRT-PCR revealed significant downregulation of BCSC genes (ACTC2, Cd44, Nanog, Pou5F1, SOX-2) and EMT markers (TGFβ1, vimentin, SMA) in cambinol-treated tumors (number = 2). (**D**) Western blot showed reduced vimentin protein across all treated groups.

To evaluate the effects of SIRT1 inhibition in vivo, iRFP labeled MDA MB 231 cells were orthotopically implanted into the mammary fat pads of Nu/Nu mice. Once tumors were established, mice were treated with vehicle control (number = 5), cisplatin (number = 5), cambinol (number = 3), or a combination of cisplatin and cambinol (number = 4). Cambinol treatment significantly inhibited tumor growth throughout the treatment period, resulting in markedly reduced tumor volumes and weights compared with control mice (*p* < 0.05; Figure 4A). Cisplatin monotherapy initially suppressed tumor growth but was followed by rapid tumor growth, indicating possible acquired drug resistance to Cisplatin. However, this rapid tumor growth was not observed in the combination treatment group (Cisplatin + Cambinol), suggesting that SIRT1 inhibition delays or prevents cisplatin resistance.

Our study demonstrated a higher incidence of lymph node metastasis than hematogenous metastasis of breast cancer, with only one case of lung metastasis observed in the cisplatin group. Based on iRFP whole-body imaging and lymph node imaging, lymph node metastasis was detected in 5 of 5 control group mice (15 of 37 lymph nodes), 4 of 5 cisplatin group mice (9 of 40 lymph nodes), 3 of 4 mice in cambinol+ cisplatin (5 of 40 lymph nodes), and 0 of 3 cambinol group mice (0 of 20 lymph nodes). Furthermore, H&E staining of skin sections revealed marked lymphovascular invasion in the control group. The cisplatin and cambinol + cisplatin groups also showed lymphovascular invasion, whereas no apparent lymphovascular invasion was observed in the cambinol-treated group. These findings suggest that SIRT1 inhibition with cambinol may suppress lymphovascular invasion and lymph node metastasis in this model. (Figure 4B). iRFP imaging and histologic analyses confirmed the absence of lymphovascular invasion following SIRT1 inhibition. Gene expression analysis of tumor tissues demonstrated significant downregulation of BCSC markers (CD44, Nanog, Pou5f1, SOX-2) and EMT-associated genes (TGF β1, vimentin, SMA) in the cambinol-treated group (Figure 4C,D). Due to the limited tumor size of the xenograft mice, we did not have enough tumor tissue for further analyzing of qPCR or Western blot of Wnt pathway components, such as β-catenin, GSK3ab and DVLs. These results indicate that SIRT1 inhibition suppresses tumor growth, metastatic spread, and stemness-associated transcriptional programs in vivo.

### 2.5. SIRT1 Inhibition Attenuates Wnt/β Catenin Signaling in Breast Cancer Cells

**Figure 5:**
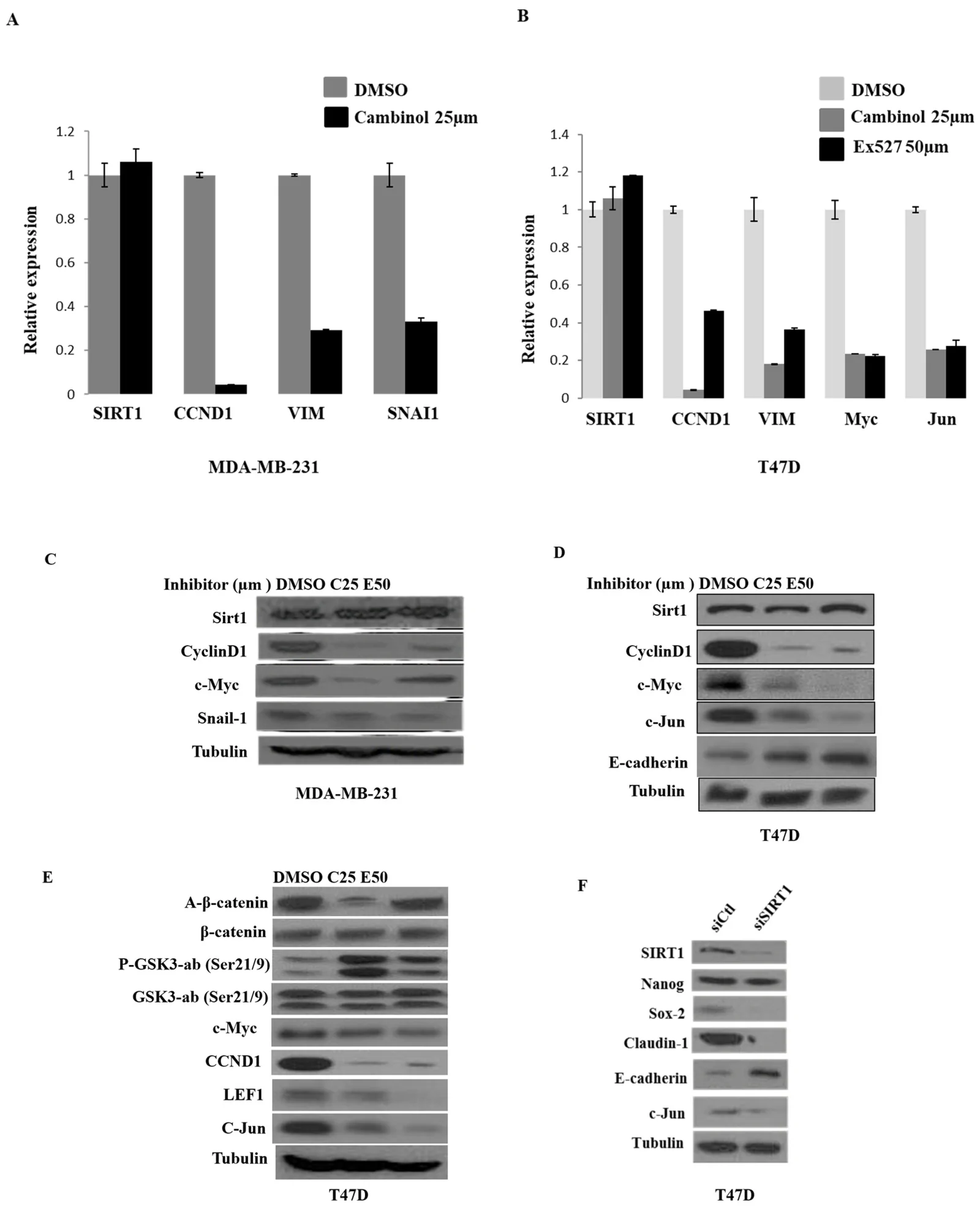
SIRT1 inhibition significantly down-regulated Wnt/β-catenin target genes in breast cancer cells. qPCR and Western blots were performed in MDA-MB-231 (**A**,**C**) and T47D (**B**,**D**,**E**) cells. (**F**) The silence of SIRT1 was performed by siRNAs. Cells were incubated with SIRT1 inhibitors. C25: cambinol (25 µM); E50: Ex527 (50 µM).

Next, we investigated whether Wnt/β-catenin signaling is involved in the response to SIRT1 inhibition. Treatment of MDA MB 231 and T47D cells with cambinol or EX 527 significantly reduced expression of canonical Wnt target genes, including CCND1 (Cyclin D1), c Myc, and Vimentin (Figure 5A–E), without altering total SIRT1 expression. These results indicate that SIRT1 inhibition suppresses the Wnt/β-catenin pathway.

To further explore whether SIRT1 is involved in the regulation of the Wnt/β-catenin pathway. We performed SIRT1 silencing with SIRT1 siRNAs. Using the breast cancer cell line T-47D, SIRT1 siRNA significantly blocked the Wnt pathway and down-regulated several stem cell markers such as Sox-2 and Nanog. EMT markers, including claudin-1 and E-cadherin, were affected (Figure 5F).

Taken together, our results indicated that SIRT1 was associated with the regulation of Wnt/β-catenin signaling; the Wnt pathway is highly activated in BCSCs and EMT of human breast cancer cell lines.

### 2.6. Wnt/β-Catenin and TGF-β Signaling Were Associated with the Promotion of Stemness and EMT in Breast Cancer Cells

**Figure 6:**
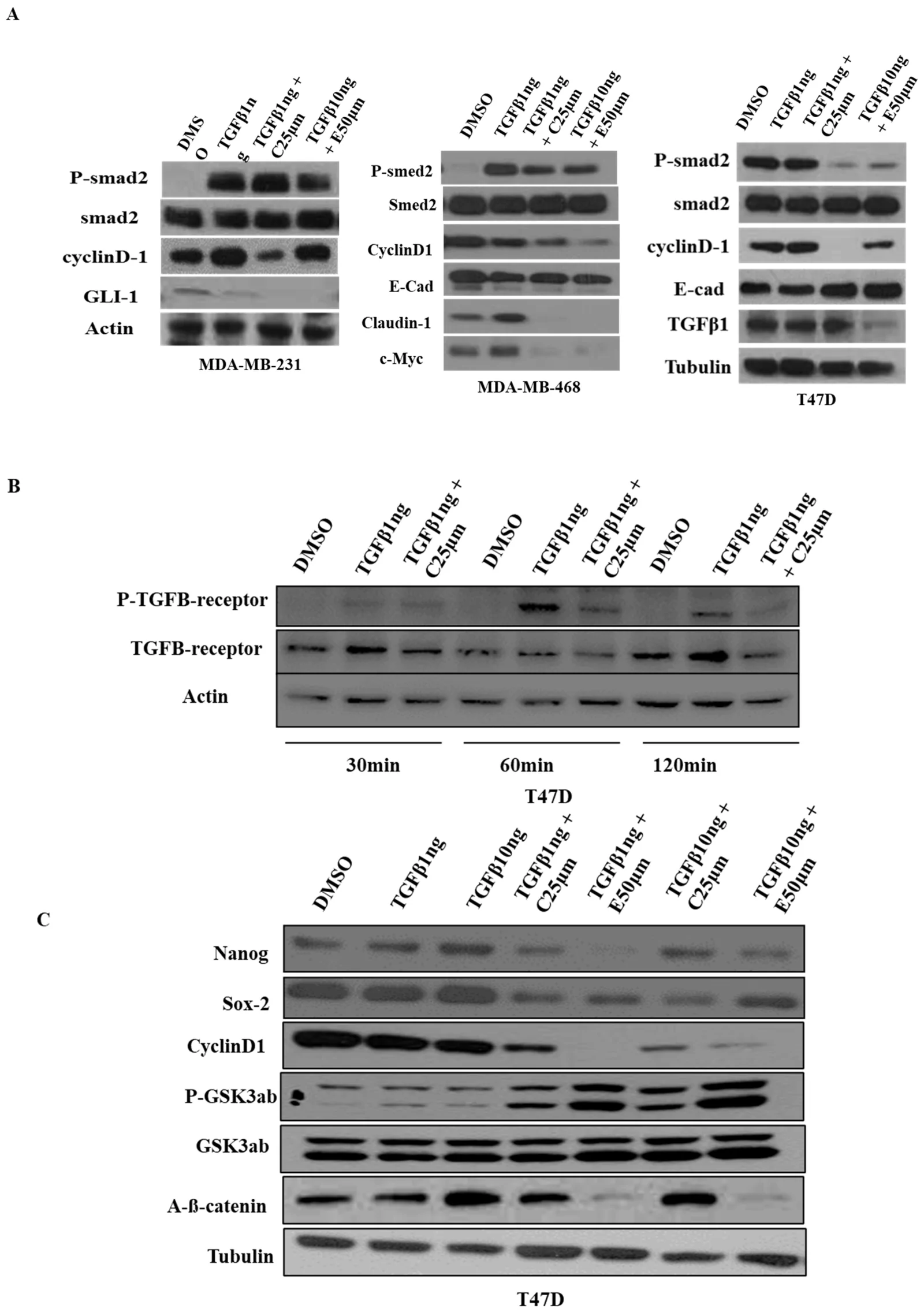
TGF-β and Wnt/β-catenin coordinated regulation of stemness and EMT genes. (**A**) SIRT1 promoted TGF-β signal and suppressed by SIRT1 inhibitors in breast cancer cells. (**B**) A time course to examine P-TGF-β receptor or TGF-β receptor at 30, 60 and 120 min in T47D cells. Western blots were performed in T47D cells (**C**) shown TGF-β and wnt/β-catenin pathways are both regulated by SIRT1 inhibition. Cells were incubated with TGF-β alone or together with SIRT1 inhibitors. C25: cambinol (25 µM); E50: Ex527 (50 µM).

Several key genes are co-regulated by both TGF-β and Wnt/β-catenin signaling, particularly those involved in EMT [41,42,43], stemness [44], invasion, and metastasis [45], and these two pathways frequently cooperate to drive aggressive tumor phenotypes [46]. TGF-β and Wnt/β-catenin signaling have been reported to interact at multiple levels, including potential transcriptional cooperation between SMAD2/3-SMAD4 and TCF/LEF–β-catenin on shared target genes. In addition, evidence from previous studies suggests that TGF-β signaling may influence β-catenin stabilization, whereas Wnt signaling may affect SMAD activity and nuclear retention. Based on these reported associations, we examined the expression of TGF-β- and Wnt/β-catenin-related genes, as well as their common downstream target genes, following TGF-β stimulation and SIRT1 inhibition in breast cancer cells. Expression of cell cycle- and proliferation-associated genes, such as Cyclin D1, as well as the EMT-related gene Claudin-1, was increased following TGF-β stimulation and suppressed by SIRT1 inhibition. In contrast, expression of the epithelial marker E-cadherin was decreased by TGF-β stimulation and restored following treatment with SIRT1 inhibitors (Figure 6A).

Activation of TGF-β signaling involves phosphorylation of the TGF-β receptor [47]. To investigate whether SIRT1 regulates TGF-β signaling, we examined the expression and activation of key components of this pathway. Phosphorylation of the TGF-β receptor increased in a time-dependent manner following TGF-β stimulation at 30, 60, and 120 min, whereas this phosphorylation was markedly suppressed by cambinol treatment in T47D cells (Figure 6B).

In T47D cells, stimulation with increasing concentrations of TGF-β (1 or 10 μg) induced dose-dependent upregulation of the stemness markers Nanog and SOX-2. This induction was markedly attenuated by treatment with either cambinol or EX-527 (Figure 6C). In addition, SIRT1 inhibition increased phosphorylation of GSK3α/β, consistent with reduced levels of active β-catenin. Together, these findings indicate that SIRT1 inhibition suppresses TGF-β- and Wnt/β-catenin-associated signaling outputs linked to EMT and stemness.

### 2.7. SIRT1 Inhibition Is Associated with DVL-3 and Wnt/β-Catenin Pathway Downregulation

Disheveled (DVL) proteins are essential cytoplasmic transducers that convey Wnt ligand engagement at Frizzled receptors to both canonical and non-canonical downstream pathways. In breast cancer, DVL isoforms-particularly DVL1 and DVL3-are frequently overexpressed [48] and promote tumor progression by stabilizing β-catenin, suppressing the β-catenin destruction complex, and enhancing transcription of oncogenic, EMT-related, and stemness-associated genes [48]. Given that DVL3 overexpression has been reported in breast carcinomas [49], we focused on characterizing its relationship with SIRT1.

**Figure 7:**
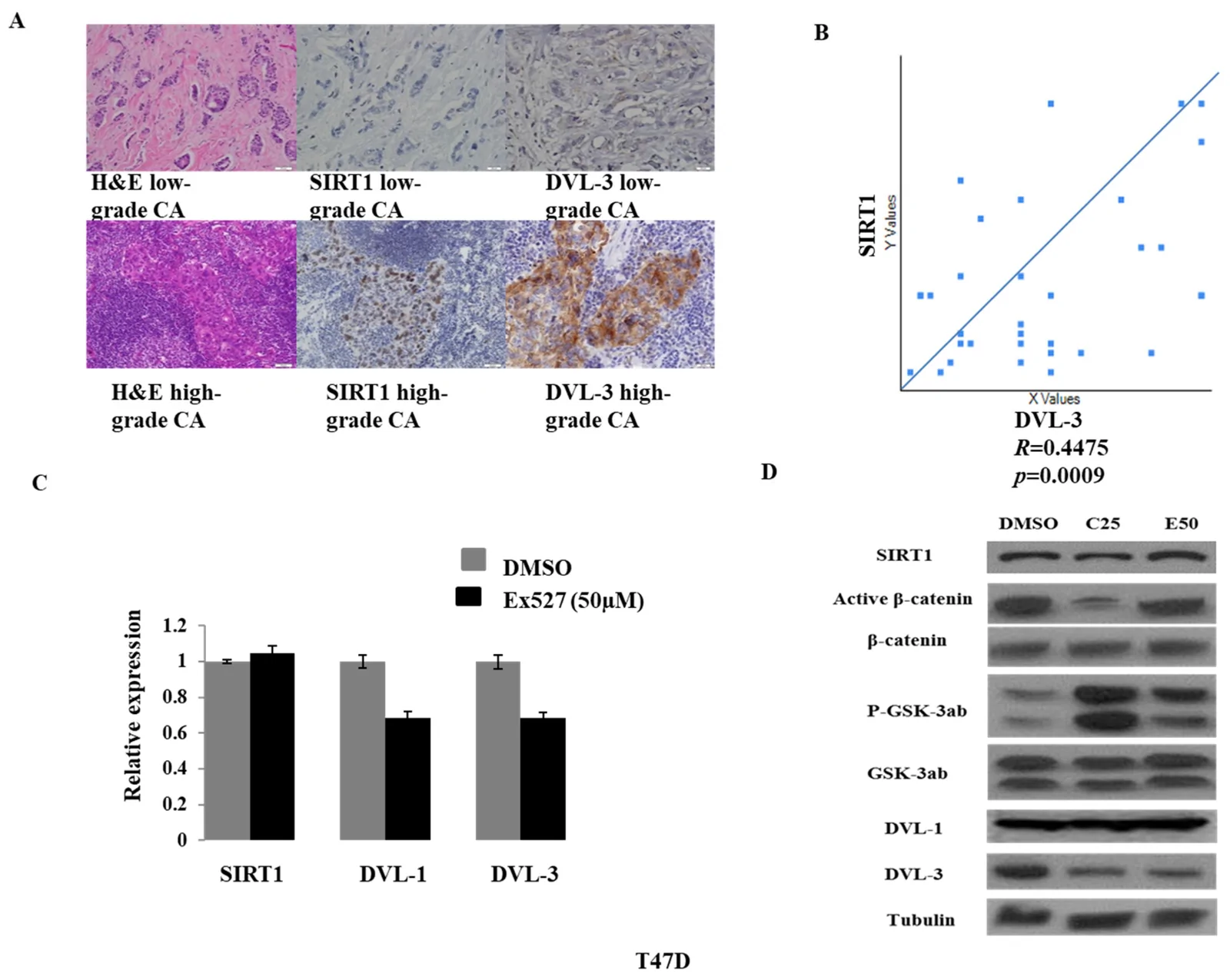
SIRT1 inhibition blocks BCSCs via the Wnt/β-catenin pathway mediated by DVL3. (**A**,**B**) Immunohistochemical staining for SIRT1, DVL3, β-catenin, and active β-catenin was performed on 32 breast cancer specimens. SIRT1 and vimentin expression levels were positively correlated with DVL3 protein expression in breast cancer tissues. Scale bar, 50 μm. The qPCR (**C**) and Western blots (**D**) were performed with T47D cells. Cells were incubated with Cambinol (25 µM) or EX527 (50 µm) for 24 h, and protein levels involved in the genes of the Wnt/β-catenin pathway were examined.

Immunohistochemistry for SIRT1, DVL3, β-catenin, and activated β-catenin was performed on 32 breast cancer specimens. SIRT1 and Vimentin expression showed a positive correlation with DVL3 protein levels (Figure 7A,B). Nuclear β-catenin accumulation was not observed, and no correlation was detected between SIRT1 and activated β-catenin, possibly due to limitations in distinguishing cytoplasmic from nuclear β-catenin when present at low levels.

Because DVL3 overexpression has been documented in breast cancer, we further examined its regulation by SIRT1. Treatment with the SIRT1-specific inhibitor EX527 reduced the mRNA levels of DVL1 and DVL3, but not SIRT1 itself (Figure 7C). Consistent with this finding, Western blot analysis showed that DVL3 protein expression was markedly decreased (DVL1 showed no change) following treatment with the SIRT1 inhibitors cambinol and EX527 in T47D cells (Figure 7D). Given the requirement of β-catenin for tumorigenic behavior in breast cancer, we hypothesized that SIRT1 inhibition would reduce active β-catenin levels. Indeed, in T47D cells, active β-catenin expression was downregulated by both cambinol and EX527, while phosphorylated GSK3α/β, the inhibitory form of GSK3α/β, was increased. Neither inhibitor affected total SIRT1 expression. Our findings suggest that DVL-3 may contribute to the association between SIRT1 and Wnt/β-catenin signaling in breast cancer stem cell maintenance, although further studies are required to establish a direct mechanistic relationship.

**Figure 8:**
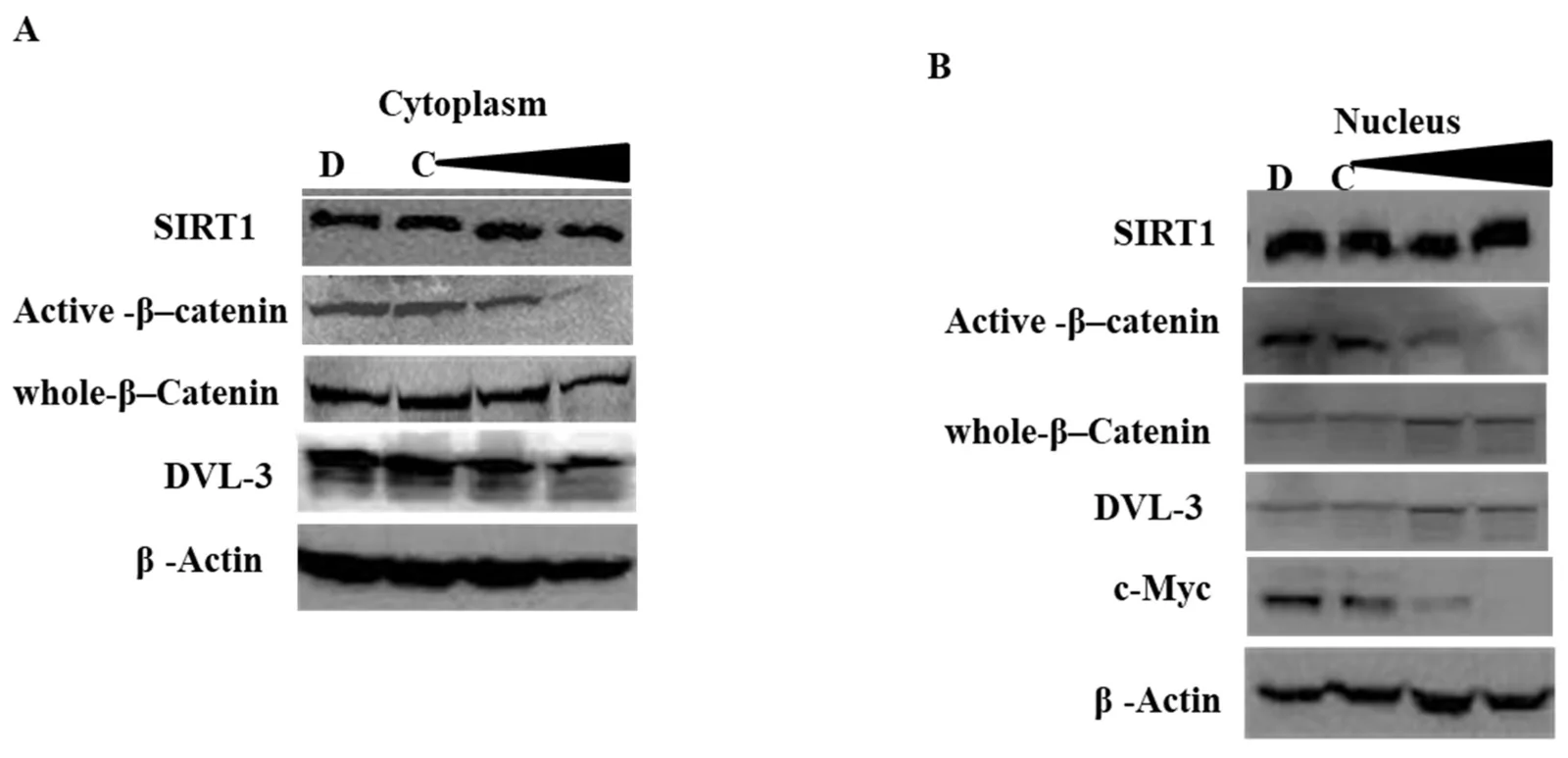
DVL3 is associated with cytoplasmic Wnt/β-catenin signaling following SIRT1 inhibition. T47D breast cancer cells were treated with DMSO or cambinol for 24 h. Equal amounts of protein (15 μg) from cytoplasmic (**A**) or nuclear (**B**) fractions were analyzed. Cytoplasmic and nuclear proteins were extracted using the NE-PER Nuclear and Cytoplasmic Extraction Reagents Kit (Thermo Fisher Scientific, Waltham, MA, USA). D, DMSO; C, cambinol.

SIRT1 is typically described as a nuclear deacetylase, whereas DVL family proteins are generally cytoplasmic; however, several studies have reported altered or even opposite subcellular localization patterns in cancer cells [50]. To clarify this in breast cancer cells, we performed cytoplasmic and nuclear fractionation followed by immunoblotting to examine SIRT1, DVL3, and β-catenin under SIRT1 inhibition. As shown in Figure 8, DVL3 localization was down-regulated by SIRT1 inhibition in cytoplasmic fractions; at the same time, DVL3 was hardly detected in the nuclear fraction, which meant it localized primarily in the cytoplasm, whereas β-catenin levels progressively decreased with increasing concentrations of cambinol. Similar results were observed in MDA-MB-231 cells. Further analysis of active versus whole β-catenin revealed that both forms of β-catenin were down-regulated by cambinol in a dose-dependent manner.

Collectively, these findings demonstrate that SIRT1 inhibition suppresses the Wnt/β-catenin pathway in breast cancer cells, characterized by reduced active and inactive β-catenin, suggesting that the anti-BCSC effects of SIRT1 inhibition may be mediated through disruption of Wnt/β-catenin signaling.

## 3. Discussion

In this study, we analyzed SIRT1 expression in 32 breast cases and found that SIRT1 was highly expressed in breast cancer (62.9%). Consistently, EMT marker expressions are associated with tumor grade, showing decreased E-cadherin and increased vimentin in high-grade breast cancer. Pearson correlation analysis revealed a significant positive correlation between SIRT1 and vimentin expression, suggesting that SIRT1 may promote breast cancer progression through enhancement of BCSC and EMT phenotypes.

Inhibition of SIRT1 significantly reduced ALDH activity and the CD44^+^/CD24^−/low^ population in breast cancer cells. In parallel, key stemness-associated genes downstream of the Wnt/β-catenin and TGF-β pathways, including SOX-2 and Nanog, were markedly down-regulated at both mRNA and protein levels. EMT-related genes were also altered, with Claudin-1 down-regulated and E-cadherin up-regulated following SIRT1 inhibition. Mammosphere assays further demonstrated that SIRT1 inhibition significantly impaired mammosphere formation, indicating reduced self-renewal capacity of BCSCs. The invasion assay revealed that Sirt1 inhibition suppressed invasion in breast cancer cells. Collectively, these data indicate that SIRT1 inhibition suppresses BCSC maintenance, consistent with previous reports describing SIRT1 as a positive regulator of cancer stem cells in breast cancer [27,51,52,53], highlighting its critical role in preserving CSC characteristics.

SIRT1 has been strongly expressed in prostate cancer [54], leukemia lymphoblasts [55,56], hepatic cancer [37,57,58], colorectal cancer [59], and HPV-infected cervical cancer [60], and acted as a tumor promoter. To date, most studies suggest that SIRT1 acts as a tumor promoter in breast cancer. For instance, a study investigating SIRT1 polymorphisms in 541 breast cancer patients and 439 healthy controls found significantly higher SIRT1 expression in the patient group. In addition, the frequency of single nucleotide polymorphisms (SNPs) in the SIRT1 gene was elevated in patients and was associated with increased breast cancer risk and poorer prognosis in Egyptian women [61]. On the other hand, some studies described that SIRT1 expression is reduced in triple-negative breast cancer (TNBC) cells, suggesting that reduced SIRT1 may contribute to TNBC aggressiveness through secretome-mediated mechanisms [62]. Simic et al. described that SIRT1 reduces EMT in cancer and fibrosis by deacetylating Smad4 and repressing the effect of TGF-β signaling [63]. Thus, SIRT1 exhibits context-dependent dual roles, acting either as a tumor suppressor or a tumor promoter depending on cell type, genetic background, and oncogenic environment [64].

Our xenograft model with nude mice and MDA-MB-231 cells showed that the SIRT1 inhibitor cambinol decreased breast cancer tumor growth and blocked cancer metastasis. The BCSC-associated genes Nanog, Sox-2 and CD44 were greatly down-regulated by SIRT1 inhibition. Our data supported that SIRT1 inhibitors block EMT and reduce the differentiation of stem-like cells in xenograft tumor tissue.

One interesting observation was that SIRT1 inhibition not only suppressed tumor growth but also appeared to overcome chemotherapy resistance in mice. After 15 days of treatment, tumors in the cisplatin-only group lost responsiveness to Cisplatin, and they started to grow rapidly and almost reached the size of those in the control group, indicating the development of drug resistance. However, in the cambinol + cisplatin group, resistance to cisplatin was observed, which meant SIRT1 inhibition reversed tumor cell cisplatin resistance. Cisplatin resistance in cancer has been frequently reported [57,65,66] with CSCs, contributing to one of the major mechanisms. However, the precise timeline for the emergence of cisplatin resistance remains unclear. Currently, there is definitive evidence showing that SIRT1 inhibition reverses cisplatin resistance in breast cancer [67]. Additionally, several studies have demonstrated that SIRT1 promotes cisplatin resistance in other malignancies, including bladder cancer [68], ovarian cancer [69], and endometrial cancer [70]. SIRT1 influences redox balance, cellular senescence, DNA repair, and stress-response pathways, mechanisms closely associated with chemoresistance and the maintenance of cancer stem cells (CSCs) [67].

In our in vivo study, we observed that the cambinol-treated group exhibited the greatest degree of tumor suppression. Because cambinol does not have an established standard in vivo dose, we referred to published studies in which 100 µM or 100 mg/kg per mouse was commonly used [66,67], and 100 mg/kg was reported as the highest tolerated dose without causing body-weight loss [68]. However, significant toxicity was observed during the study, and two of the five mice in the cambinol-treated group likely died of drug toxicity. A limitation of this study is the relatively small sample size of the xenograft experiments (approximately number = 5 per group), which limits statistical power and the ability to draw definitive conclusions regarding metastatic potential and therapeutic resistance. Although the xenograft model provides preliminary in vivo evidence that SIRT1 inhibition suppresses tumor growth and may influence therapeutic response, these findings should be interpreted cautiously. Larger cohorts and additional in vivo models, including models that more effectively recapitulate metastatic dissemination and treatment resistance, will be needed to validate these observations. The potential safety and toxicity of cambinol should also be considered when interpreting its in vivo therapeutic effects. Although cambinol has demonstrated antitumor activity in preclinical models and a dose of 100 mg/kg has been reported without apparent toxicity in some mouse studies, the therapeutic window and long-term safety of SIRT1 inhibition remain incompletely characterized. Therefore, our xenograft findings provide preliminary evidence of antitumor activity but do not establish the safety or clinical applicability of cambinol. Further studies are needed to define the pharmacokinetics, dose-dependent toxicity, tolerability, and therapeutic window of SIRT1 inhibitors.

Recent studies have shown that SIRT1 regulates BCSCs by activating the Wnt/β-catenin pathway [17,66], a pathway well known for promoting and maintaining cancer stem cells during tumorigenesis [37,66]. In our work, inhibition of SIRT1 led to a clear downregulation of Wnt/β-catenin target genes, including Cyclin D1 and c-Myc, as well as a reduction in active β-catenin levels. The role of the Wnt/β-catenin pathway in promoting EMT has been described in several cancers, and β-catenin has been reported to positively regulate vimentin expression [9,11,71]. Consistent with these findings, our results demonstrated that the SIRT1 inhibitors cambinol or EX527 markedly down-regulated vimentin while up-regulating E-cadherin.

GSK-3 is a key regulator in the Wnt/β-catenin pathway, negatively controlling β-catenin through phosphorylation-mediated ubiquitination [43]. Therefore, we examined the phosphorylation status of GSK3α/β (Ser21/9) and its total protein levels following treatment with cambinol or EX527. Our results showed that phosphorylated GSK3α/β (Ser21/9) was increased upon SIRT1 inhibition, suggesting that GSK3 may transfer its phosphate group to β-catenin, promoting β-catenin degradation when SIRT1 is inhibited. Furthermore, silencing SIRT1 in breast cancer cells confirmed that SIRT1 was associated with the regulation of Wnt/β-catenin signaling.

The Wnt/β-catenin and TGF-β signaling pathways are highly interconnected and cooperate to promote tumor progression, stemness, and EMT in cancer, including breast cancer [72,73]. In our study, inhibition of SIRT1 altered common downstream targets of these pathways, including stem cell genes and EMT-related genes, which were either down- or up-regulated. Mechanistically, TGF-β can enhance Wnt/β-catenin signaling by increasing β-catenin stability, promoting its nuclear translocation, and facilitating β-catenin/TCF transcriptional complex formation [74]. While Wnt signaling can potentiate TGF-β activity by boosting Smad2/3 transcriptional output and shared EMT/stemness targets [46]. Together, these pathways co-activate key transcription factors such as Snail, Slug, ZEB1, ZEB2, and Twist, driving loss of epithelial identity and increased motility [42], and they enhance core stem cell markers including SOX-2, NANOG, OCT4, and CD44 [75]. Their convergence further promotes invasion through coordinated induction of MMPs and cytoskeletal remodeling genes. This crosstalk is mediated through GSK-3β inhibition, SMAD2/3–SMAD4 complexes, and stabilized β-catenin, which integrate signals to amplify EMT, invasiveness, and CSC phenotypes.

Our experiments demonstrated that DVL3, a key component of Wnt signaling, is highly expressed in higher-grade breast cancers and positively correlates with SIRT1 and vimentin expression in breast cancer specimens. These findings provide clear evidence that SIRT1 promotes BCSC-associated DVL3 activity in breast cancer. Holloway KR et al. [49] showed that SIRT1 forms a complex with DVL3 and β-catenin and that SIRT1 inhibition or silencing downregulates Wnt target genes such as BMP4 and Cyclin D1. Simmons GE et al. [76] also reported that Frizzled 7, a Wnt co-receptor, is positively regulated by SIRT1. Consistent with these studies, our data indicate that SIRT1 functions as a cofactor to enhance canonical Wnt/β-catenin signaling.

The canonical Wnt/β-catenin pathway activates target genes including c-Myc, Nanog, and SOX-2, which support BCSC self-renewal and tumor development [12,19]. Because our data showed activation of these genes in breast cancer tissues and cell lines, we focused on the canonical pathway. Disheveled proteins (DVL1-3) are essential components of Wnt/β-catenin signaling [77]. DVLs interact with Axin, GSK3, and β-catenin to form a regulatory complex. When deacetylated by SIRT1, this complex becomes more stable [49]. Activated DVL further interacts with Frat, a GSK3-binding protein, drawing GSK3 away from the complex. This increases the pool of free β-catenin, allowing its translocation into the nucleus to initiate transcription [77,78].

In our study, SIRT1 expression was positively correlated with DVL3 in breast cancer specimens with significant differences, and DVL3 mRNA and protein levels were downregulated after SIRT1 inhibition. These findings suggest that DVL3 may be involved in the regulation of Wnt/β-catenin signaling downstream of SIRT1 and may contribute to the maintenance of BCSC properties. This is consistent with prior findings that SIRT1 deacetylates DVL proteins, including DVL3 lysines located in the DIX domain. SIRT1 also deacetylates nuclear DVL3, thereby facilitating β-catenin–TCF complex formation and transcriptional activation of Wnt target genes [49,78]. The reduced nuclear β-catenin observed after SIRT1 inhibition in our study (Figure 7) is explained by this mechanism. Evidence for SIRT1-DVL interactions promoting Wnt/β-catenin signaling has also been reported in colorectal and breast cancers [79]. In our experiment, the DVL1protein level remained unchanged; we could not detect the positive correlation between the expression of Sirt1 and DVL1, suggesting Sirt1 was not involved in DVL1 regulation in this study.

It has been reported that SIRT1-mediated deacetylation of DVLs occurs not only in the cytoplasm but also in the nucleus [79]. Consistent with this, our findings suggest that SIRT1 regulates DVL3 through post-translational mechanisms in both compartments. Like the established SIRT1/β-catenin regulatory mechanism [37], we propose that SIRT1 deacetylates DVL3 in the cytoplasm, thereby stabilizing the DVL3/Axin/β-catenin complex, increasing free β-catenin, and preventing its degradation. Concurrently, SIRT1-mediated deacetylation of DVL3 in the nucleus promotes the formation and stabilization of the SIRT1/DVL3/β-catenin/TCF complex, enhancing β-catenin-dependent transcriptional activity [80].

Accumulating evidence indicates that SIRT1 exhibits context-dependent and dual roles in cancer biology. On one hand, SIRT1 has been described as a putative tumor suppressor due to its anti-inflammatory functions. SIRT1 negatively regulates inflammation-associated carcinogenesis by deacetylating and inhibiting key proinflammatory transcription factors, including NF-κB, AP-1, and signaling downstream of TNF-α, thereby reducing chronic inflammation and genomic stress that favor tumor initiation [24,63,81]. On the other hand, substantial evidence supports an oncogenic role for SIRT1. Elevated SIRT1 expression has been reported in multiple malignancies, including prostate cancer [54], leukemia and lymphoblasts [55,56], colorectal cancer [82], cervical cancer [60], and breast cancer [25,33,51]. These observations suggest that SIRT1 contributes to tumor progression, therapy resistance, and cancer stem cell maintenance, highlighting its importance in tumorigenesis and cancer evolution [83,84].

Mechanistically, SIRT1 exerts its effects through deacetylation of both histone and non-histone substrates. In the nucleus, histone deacetylation by SIRT1 promotes chromatin compaction, leading to transcriptional repression of target genes, including tumor suppressors [25]. In contrast, the consequences of non-histone protein deacetylation are highly substrate- and context-specific. SIRT1 modulates the activity, stability, localization, and protein–protein interactions of numerous non-histone targets such as p53, FOXO proteins, β-catenin, and DVL proteins, resulting in diverse and sometimes opposing biological outcomes [21,85].

Recent concepts emphasize that the functional outcome of SIRT1 activity is highly context-dependent, influenced by tumor type, genetic background, metabolic state, subcellular localization, and the availability of specific substrates. This complexity provides a plausible explanation for the dual tumor-suppressive and tumor-promoting roles of SIRT1 in cancer [21,22,83].

Taken together, our results demonstrate an important role for SIRT1 in BCSCs and suggest that SIRT1 positively regulates cancer stem-like cell properties in breast cancer. The consistent effects observed with cambinol and the more selective SIRT1 inhibitor EX-527 provide supportive evidence for the involvement of SIRT1 in regulating these phenotypes. These findings highlight the biological significance of SIRT1 and support its potential as a therapeutic target in breast cancer. However, the toxicity, safety, and optimal therapeutic dose of SIRT1 inhibitors require further evaluation in preclinical and clinical studies. Although cambinol was used as an SIRT1 inhibitor in our study, its reported activity against SIRT2 and nSMase2 should be considered when interpreting our findings. Therefore, the potential contribution of SIRT2, nSMase2, or other off-target effects of cambinol cannot be completely excluded based on the current experiments. Further studies using additional SIRT1-selective approaches and complementary genetic strategies will be important to further confirm the specific role of SIRT1 in breast cancer stem-like cell properties and its therapeutic potential.

## 4. Materials and Methods

### 4.1. Breast Cancer Specimens

Tumor tissues from 32 breast cancer patients, representing various stages, grades, and subtypes, were collected via surgery or biopsy. Patient consent was waived due to it being a retrospective study. Additionally, 2 normal breast tissue samples from the corresponding patients were obtained as controls. The Institutional Review Board (IRB, HSC-MS-13-0023) of The University of Texas Health was approved before the study. Paraffin-embedded slides were prepared from these specimens for subsequent immunohistochemistry analysis.

### 4.2. Immunohistochemistry (IHC)

Paraffin-embedded sectioned at 3–4 μm, mounted on charged slides, and dried at 60 °C for 1 h. Sections were deparaffinized in xylene and rehydrated through graded ethanol. Antigen retrieval was performed in citrate buffer (pH 6.0) or EDTA buffer (pH 8.0) using a steamer for 45 min, followed by cooling at room temperature for 25–30 min. Endogenous peroxidase was blocked with 3% H_2_O_2_ for 10 min, followed by 2.5% normal horse serum for 20 min. IHC was performed for SIRT1 (ab32441, Abcam, Cambridge, UK, 1:100), CD44 (NCL-CD44-2, Leica Microsystems, Wetzlar, Germany, 1:100), ALDH1A (HPA005456, Sigma-Aldrich, St. Louis, MO, USA, 1:300), SOX-2 (#3579, Cell Signaling Technology, Danvers, MA, USA, 1:100), Vimentin (Clone V9, Dako, Glostrup, Denmark, 1:100) and Nanog (#4903, Cell Signaling, 1:200) with one hour incubation at room temperature or overnight at 4 °C. Sections were then incubated with the appropriate Vectastain Elite ABC secondary antibody (PK-7200, Vector Laboratories, Burlingame, CA, USA) and ABC reagent for 30 min each at room temperature. Immunoreactivity was visualized with DAB for 2–5 min and counterstained with hematoxylin. Sections were dehydrated, cleared in xylene, and mounted. Negative controls without primary antibody and positive controls with known strong expression were included in each assay. All markers were scored blindly using the H-score method, calculated as staining intensity (0–3) × percentage of positive cells (0–100), yielding scores of 0–300. Data were analyzed using Chi-square tests and Pearson correlation coefficients.

### 4.3. Cell Lines and Culture

The breast cancer cell lines T47D (HTB-113), MDA-MB-468 (HTB-132), BT549 (HTB-122) and MDA-MB-231 (HTB-26) were obtained from ATCC (Manassas, VA, USA) and maintained at 37 °C with 5% CO_2_ in complete RPMI 1640 or DMEM medium supplemented with 10% fetal bovine serum (FBS) and 1% penicillin/streptomycin. Media were obtained from Invitrogen and FBS from Sigma-Aldrich, Cultures were refreshed every 2–3 days and trypsinized at 70–80% confluence.

### 4.4. Flow Cytometry Analysis

T47D and MDA-MB-231 cells (1 × 10^6^) were incubated with fluorescently conjugated antibodies, including anti-CD24 (clone ML5; PE) and anti-CD44 (clone G44-26; APC) from BD Biosciences, San Jose, CA, USA. Samples were analyzed using an EasyCyte flow cytometer (Guava FCM, Millipore, Billerica, MA, USA) according to the manufacturer’s instructions.

ALDH activity was measured using the ALDEFLUOR^®^ assay kit (StemCell Technologies, Vancouver, BC, Canada). Harvested cells (6 × 10^5^/mL) were incubated with the ALDEFLUOR^®^ substrate in a 37 °C water bath for 45 min. The substrate BODIPY-aminoacetaldehyde (BAAA) is converted by ALDH into a fluorescent product. As a negative control, an aliquot of cells was treated with 1.5 mM diethylaminobenzaldehyde (DEAB), a specific ALDH inhibitor. Cells were then analyzed by flow cytometry.

### 4.5. Mammosphere

Mammospheres are clusters of cells exhibiting self-renewal and proliferative properties similar to stem cells [25]. Mammosphere culture and differentiation were performed as described by [2], with modifications by [1]. T47D cells were maintained in RPMI medium containing 10% fetal bovine serum at 37 °C with 5% CO_2_. For sphere formation, single-cell suspensions were transferred to poly-2-hydroxyethylmethacrylate-coated 6-well plates at 1000 cells/mL in serum-free DMEM supplemented with 1% L-glutamine, 1% penicillin/streptomycin, 30% F12, 2% B27, 20 ng/mL EGF, and 20 ng/mL FGFb. The medium contained 0.5% methylcellulose to prevent cell aggregation. After 7 days, mammospheres were collected by gentle centrifugation (200× *g*), enzymatically dissociated with 1:1 trypsin/DMEM at 37 °C for 5 min, and mechanically dissociated through a 25G needle (6 strokes). Single cells were reseeded at 1000 mL for subsequent passages. Mammospheres with diameters > 75 μm were counted to quantify sphere formation. For differentiation, individual mammospheres were cultured for 10–12 days, photographed, and colonies > 75 μm were quantified [86].

### 4.6. Cell Invasion Assay

Cell invasion assays were performed using Matrigel-coated 6-Transwell inserts [87]. BT-549, MDA-MB-231, and MDA-MB-468 cells (2 × 10^5^ cells/well) in serum-free DMEM were seeded into the upper chambers of Matrigel™-coated inserts (6-well plates, 8-μm pore size; BD BioCoat, BD Biosciences, San Jose, CA, USA). The lower chambers were filled with DMEM containing 10% FBS. After 24 h of incubation at 37 °C in 5% CO_2_, non-invasive cells on the upper surface of the membrane were removed with a cotton swab. Cells that had invaded through the Matrigel and membrane were fixed and stained with Diff-Quik (IMEB Inc., San Marcos, CA, USA). Invaded cells were quantified by counting five random fields per insert under a phase-contrast microscope. Three independent inserts were analyzed for each condition.

### 4.7. Quantitative RT-PCR

Total RNA from T47D and MDA-MB-231 cells was extracted using the RNeasy Plus Universal Mini Kit (Cat. 73404; QIAGEN, Hilden, Germany) and reverse-transcribed to cDNA using iScript Reverse Transcription Supermix (Cat. 170-8840; Bio-Rad Laboratories, Hercules, CA, USA). The RT program was 25 °C for 5 min, 42 °C for 30 min, and 85 °C for 5 min. Quantitative PCR was performed using a Real-Time PCR Detection System (Bio-Rad Laboratories, Hercules, CA, USA) with CFX Manager Software version 3.1. and gene-specific primers. HPRT1 and TBP were used as housekeeping genes for normalization in all qRT-PCR experiments. Each 20 μL reaction contained 1 μL cDNA (10 ng), 10 μL SsoAdvanced Universal SYBR Green Supermix (Cat. 172-5271; Bio-Rad Laboratories), and 8 μL nuclease-free water. PCR cycling consisted of 95 °C for 2 min, followed by 40 cycles of 95 °C for 5 s and 60 °C for 30 s, followed by melting curve analysis. PCR, RT, RNA quality, and genomic DNA contamination controls were included. Primers for SIRT1, Nanog, SOX-2, DVL1, DVL3, CCND1, c-Myc, c-Jun, GSK3α, and GSK3β were purchased from Bio-Rad. Relative gene expression was calculated using the 2^−ΔΔCt^ method and normalized to HPRT1 and TBP. Results are presented as mean ± SD. Statistical significance was determined using Student’s *t*-test, with *p* < 0.05 considered statistically significant.

### 4.8. Western Blot

Cells were washed with PBS and lysed on ice in 1× lysis buffer (Cell Signaling Technology) containing 20 mM Tris-HCl (pH 7.5), 150 mM NaCl, 1 mM Na_2_EDTA, Cell Signaling Technology 1 mM EGTA, 2.5 mM sodium pyrophosphate, 1 mM β-glycerophosphate, 1 mM Na_3_VO_4_, 1% Triton X-100, and protease/phosphatase inhibitors (Calbiochem, San Diego, CA, USA). Protein samples (10–30 μg) were separated on 8–12% SDS-PAGE gels and transferred to 0.2-μm PVDF membranes (Bio-Rad Laboratories). Membranes were incubated with primary antibodies against SOX-2 (#3579), Nanog (#4903), p-GSK-3α/β (#9331), c-Myc (#5605), β-actin (#4970), and β-catenin (#8480) (Cell Signaling Technology); Cyclin D1 (M3642, Dako, Glostrup, Denmark); SIRT1 (ab32441, Abcam); DVL-1 (SC-8025), DVL-2 (SC-13974), DVL-3 (SC-271295), and tubulin (SC-23950) (Santa Cruz Biotechnology); and GSK-3α/β (05-903, Millipore, Billerica, MA, USA). After incubation with HRP-conjugated secondary antibodies (GE Healthcare, Chicago, IL, USA), protein signals were detected using SuperSignal West Pico or Pierce ECL substrates.

### 4.9. SIRT1 siRNA Silencing

Cells were seeded in 6-well plates in antibiotic-free growth medium and incubated overnight. Control siRNA or siSIRT1 (Cat. 12241; Cell Signaling Technology; sc-40986, Santa Cruz Biotechnology, Dallas, TX, USA was diluted in 500 µL of serum-free DMEM, and 5 µL of Lipofectamine 2000 was diluted in 500 µL of serum-free DMEM. The diluted siRNA and Lipofectamine solutions were combined, mixed, and added to cells. After 4–6 h, 1 mL of medium with 10% FBS was added. Cells were incubated at 37 °C with 5% CO_2_ for 48 h before assays for SIRT1 knockdown efficiency.

### 4.10. Xenograft Mice Experiment

The animal study protocol was approved by both the University of Texas Health Science Center at Houston (UTHSC-Houston; Protocol No. HSC-AWC-13-017) and the Department of Defense (DOD) Animal Care and Use Committees. A total of 20 eight-week-old female athymic Nu/Nu mice were purchased from Charles River Laboratories (Wilmington, MA, USA). No formal sample size calculation was performed. Sample sizes (number = 5 per group) were selected based on previous experience with similar xenograft studies and ethical considerations to minimize animal use while maintaining adequate statistical power. The animals were obtained from an accredited commercial supplier and maintained under specific pathogen-free (SPF) conditions in accordance with institutional guidelines. Mice were housed under identical environmental conditions with free access to food and water. All healthy mice meeting the predefined criteria were included. No predefined exclusion criteria were applied unless animals developed unexpected illnesses unrelated to the experiment. All treatments and measurements were performed using standardized procedures to minimize potential confounding factors.

A mammary fat pad injection model was employed to mimic human breast cancer and allow assessment of potential lymph node metastasis. MDA-MB-231 cells were selected because of their well-established high metastatic capacity compared with other breast cancer cell lines.

Each mouse was inoculated with 1 × 10^6^ iRFP-labeled MDA-MB-231 cells into the right fourth (4th) mammary fat pad. Tumors were allowed to establish for three weeks until they became palpable. Mice were then randomly assigned to four treatment groups (number = 5 per group). Each treatment group was housed in a separate cage and received intraperitoneal injections twice weekly. The treatment groups were as follows: (1) control, 200 μL saline; (2) cambinol, 0.8 mg cambinol in 200 μL saline; (3) cisplatin, 0.075 mg cisplatin in 200 μL saline (approximately 3 mg/kg); and (4) combination treatment, 0.8 mg cambinol plus 0.075 mg cisplatin in 200 μL saline. Before each treatment, mice were examined for general health, and body weight was recorded. Any mouse that died before the planned study endpoint was excluded from the final analysis, provided that the predetermined sample size for the experiment was maintained. On day 22 after treatment initiation, all remaining mice were euthanized, and tumors were excised, measured for volume and weight, and collected for subsequent RNA and protein extraction for downstream analyses. Animals that died before the planned study endpoint were excluded from the final analysis. No other animals or data points were excluded.

## 5. Conclusions

In conclusion, our findings indicate that SIRT1 contributes to the maintenance of breast cancer stem cell-associated phenotypes and malignant behaviors, including stemness, EMT, invasion, metastasis, and therapeutic resistance. Inhibition of SIRT1 reduced these properties in breast cancer cells and xenograft models and was accompanied by decreased DVL3 expression and attenuation of Wnt/β-catenin signaling. These findings identify a potential link between SIRT1 and the DVL3/Wnt/β-catenin signaling axis in the regulation of BCSC-associated properties and provide a basis for further investigation of SIRT1-targeted strategies in breast cancer.

## Figures and Tables

**Table 1:** Clinical characteristics of the breast cancer patients and SRRT1 expressions.

Clinical Factor	Case (%)	Sirt1 Overexpression (%)	*p* Value
Histological type, n (%)	35 (100%)	22 (62.9)	
IDC	28 (84.8)	18 (64.3)	
ILC	5 (15.2)	3 (60.0)	IDC vs. ILC *p* = 0.27
Other	2 (3.0)	1 (50)	
Grade, n (%)	33 (100%)	21 (63.6)	
I	5 (15.2)	3 (14.3)	
II	11 (33.3)	6 (28.6)	
III	17 (51.5)	12 (57.1)	G1 + G2 vs. G3 *p* = 0.046
AJCC stage, n (%)	33 (100%)	21 (63.6)	
1	20 (60.6)	12 (60.0)	
2	10 (30.3)	7 (70.0)	
3	2 (6.1)	2 (100.0)	T1 vs. T2 + T3 *p* = 0.9
unknown	1 (3.0)	0 (0.0)	
Axillary lymph node status, n (%)	33 (100%)	17 (51.5%)	
Positive	14 (42.4)	6 (42.3)	
Negative	19 (57.6)	11 (57.9)	Pos vs. Neg *p* = 0.28
Hormone status, n (%)	33 (100%)	21 (63.6)	
ER+	19 (57.6)	11 (57.9)	
HER2+	5 (15.2)	4 (80.0)	Er+ vs. Her2+ *p* = 0.3
Triple negative	8 (24.2)	6 (75.0)	Er+ vs. triple negative *p* = 0.7
Unknown	1 (3.0)	0 (0.0)	

## Data Availability

The original contributions presented in this study are included in the article. Further inquiries can be directed to the corresponding authors.
